# Prophylactic abdominal drainage or no drainage after distal pancreatectomy (PANDORINA): a binational multicenter randomized controlled trial

**DOI:** 10.1186/s13063-022-06736-5

**Published:** 2022-09-24

**Authors:** F. L. Vissers, A. Balduzzi, E. A. van Bodegraven, J. van Hilst, S. Festen, M. Abu Hilal, H. J. Asbun, J. S. D. Mieog, B. Groot Koerkamp, O. R. Busch, F. Daams, M. Luyer, M. De Pastena, G. Malleo, G. Marchegiani, J. Klaase, I. Q. Molenaar, R. Salvia, H. C. van Santvoort, M. Stommel, D. Lips, M. Coolsen, C. Bassi, C. van Eijck, M. G. Besselink, for the Dutch Pancreatic Cancer Group

**Affiliations:** 1 Department of Surgery, Amsterdam UMC, location University of Amsterdam, Amsterdam, the Netherlands; 2 Cancer Center Amsterdam, Amsterdam, the Netherlands; 3 Department of Surgery, Pancreas Institute, Verona University Hospital, Verona, Italy; 4 Department of Surgery, OLVG, Amsterdam, the Netherlands; 5 Department of Surgery, University Hospital Southampton NHS Foundation Trust, Southampton, UK; 6 Department of Surgery, Poliambulanza Hospital Brescia, Brescia, Italy; 7 Division of Hepatobiliary and Pancreas Surgery, Miami Cancer Institute, Miami, USA; 8 Department of Surgery, LUMC, Leiden, the Netherlands; 9 Department of Surgery, Erasmus MC, Rotterdam, the Netherlands; 10 Department of Surgery, Catharina Hospital, Eindhoven, the Netherlands; 11 Department of Surgery, University Medical Center Groningen, Groningen, the Netherlands; 12 Department of Surgery, Regional Academic Cancer Center Utrecht, University Medical Center Utrecht, Utrecht, the Netherlands; 13 Department of Surgery, Regional Academic Cancer Center Utrecht, St Antonius Hospital Nieuwegein, Utrecht, the Netherlands; 14 Department of Surgery, Radboud UMC, Nijmegen, the Netherlands; 15 Department of Surgery, Medisch Spectrum Twente, Enschede, the Netherlands; 16 Department of Surgery, Maastricht Universitair Medisch Centrum, Maastricht, the Netherlands

## Abstract

**Background:**

Prophylactic abdominal drainage is current standard practice after distal pancreatectomy (DP), with the aim to divert pancreatic fluid in case of a postoperative pancreatic fistula (POPF) aimed to prevent further complications as bleeding. Whereas POPF after pancreatoduodenectomy, by definition, involves infection due to anastomotic dehiscence, a POPF after DP is essentially sterile since the bowel is not opened and no anastomoses are created. Routine drainage after DP could potentially be omitted and this could even be beneficial because of the hypothetical prevention of drain-induced infections (Fisher, Surgery 52:205–22, 2018). Abdominal drainage, moreover, should only be performed if it provides additional safety or comfort to the patient. In clinical practice, drains cause clear discomfort. One multicenter randomized controlled trial confirmed the safety of omitting abdominal drainage but did not stratify patients according to their risk of POPF and did not describe a standardized strategy for pancreatic transection. Therefore, a large pragmatic multicenter randomized controlled trial is required, with prespecified POPF risk groups and a homogeneous method of stump closure.

The objective of the PANDORINA trial is to evaluate the non-inferiority of omitting routine intra-abdominal drainage after DP on postoperative morbidity (Clavien-Dindo score ≥ 3), and, secondarily, POPF grade B/C.

**Methods/design:**

Binational multicenter randomized controlled non-inferiority trial, stratifying patients to high and low risk for POPF grade B/C and incorporating a standardized strategy for pancreatic transection. Two groups of 141 patients (282 in total) undergoing elective DP (either open or minimally invasive, with or without splenectomy). Primary outcome is postoperative rate of morbidity (Clavien-Dindo score ≥ 3), and the most relevant secondary outcome is grade B/C POPF. Other secondary outcomes include surgical reintervention, percutaneous catheter drainage, endoscopic catheter drainage, abdominal collections (not requiring drainage), wound infection, delayed gastric emptying, postpancreatectomy hemorrhage as defined by the international study group for pancreatic surgery (ISGPS) (Wente et al., Surgery 142:20–5, 2007), length of stay (LOS), readmission within 90 days, in-hospital mortality, and 90-day mortality.

**Discussion:**

PANDORINA is the first binational, multicenter, randomized controlled non-inferiority trial with the primary objective to evaluate the hypothesis that omitting prophylactic abdominal drainage after DP does not worsen the risk of postoperative severe complications (Wente etal., Surgery 142:20–5, 2007; Bassi et al., Surgery 161:584–91, 2017). Most of the published studies on drain placement after pancreatectomy focus on both pancreatoduodenectomy and DP, but these two entities present are associated with different complications and therefore deserve separate evaluation (McMillan et al., Surgery 159:1013–22, 2016; Pratt et al., J Gastrointest Surg 10:1264–78, 2006). The PANDORINA trial is innovative since it takes the preoperative risk on POPF into account based on the D-FRS and it warrants homogenous stump closing by using the same graded compression technique and same stapling device (de Pastena et al., Ann Surg 2022; Asbun and Stauffer, Surg Endosc 25:2643–9, 2011).

**Supplementary Information:**

The online version contains supplementary material available at 10.1186/s13063-022-06736-5.

## Background

### Clinical background

The debate on routine prophylactic abdominal drainage after partial pancreatectomy has been ongoing for decades [1, 2]. Most series combine outcomes of pancreatoduodenectomy and distal pancreatectomy (DP) or focus on pancreatoduodenectomy only. There is, however, an essential difference between POPF in DP and pancreatoduodenectomy. Whereas POPF after pancreatoduodenectomy, by definition, involves infection due to anastomotic dehiscence between pancreas and small bowel, a POPF after DP is, by definition, initially sterile since no anastomoses are created. Prophylactic abdominal drainage after DP could therefore also introduce bacteria and cause drain-induced infections [3].

Recent reviews underline the need of data regarding abdominal drainage in DP [4–6]. In high-volume centers, DP has a very low mortality (0–2%) but the morbidity rate remains high (24 to 56%) with postoperative pancreatic fistula (POPF) as most common complication (0% to 61%) and cause of further complications [7–14]. Unfortunately, the risk of POPF after DP has proven difficult to predict [15]. Prophylactic abdominal drainage after DP allows for evacuation of pancreatic fluid, in case of POPF, but a drain could theoretically also induce infection, especially when left in place for prolonged periods [6, 16] or even bleeding due to vascular erosion [1]. In patients with a leak at the pancreatic transection margin, without a drain, a sterile and asymptomatic pseudocyst (i.e., no infected collection requiring no intervention) could occur [17].

A randomized controlled trial (RCT) is mandatory to determine whether patients are better off without the use of prophylactic abdominal drainage after DP.

### Need for a randomized trial

Current published series on prophylactic abdominal drainage after DP are mostly retrospective, only one multicenter randomized trial is available and this did not stratify patients according to their risk of POPF and did not use a standardized technique of transecting the pancreas [2]. It could be that outcomes of prophylactic abdominal drainage differ between patients with low and high risk of POPF after DP.

PANDORINA is a binational multicenter, randomized controlled non-inferiority trial with the primary objective to evaluate the hypothesis that omitting prophylactic abdominal drainage after DP does not worsen the risk of postoperative Clavien-Dindo score ≥ 3 complications (e.g., reinterventions, catheter drainage, wound infections, postoperative pancreatic hemorrhage, feeding tube placement) [18, 19].

## Methods

### Study design

Patients undergoing elective DP will be randomly allocated in a 1:1 ratio to no prophylactic abdominal drainage or prophylactic abdominal drainage after surgery, stratified based on the risk of POPF. This protocol was developed according to the SPIRIT guidelines [20]. Total inclusion time of the study is planned to be 24 months from start of recruitment and the total study time 36 months. The study structure includes setup of sites (4–6 months), enrollment (24–30 months), and data analysis and reporting results (4 months).

In case of readmission related to surgery within 90 days after initial discharge, follow-up for secondary outcomes will be extended to the entire duration of readmission.

### Study population

Adult patients with an indication for elective DP, with or without splenectomy, minimally invasive or open, for any indication.

### Inclusion criteria

In order to be eligible to participate to the study, a patient must meet all of the following criteria:

- ° At least 18 years old;
- ° Elective indication for DP, with or without splenectomy, minimally invasive or open, with and without sparing of splenic vessels, for all indications;
- ° Fit to undergo surgery;
- ° Oral and written informed consent.

### Exclusion criteria

Patients who meet any of the following criteria will be excluded from participation in this study:

- ° Pregnancy;
- ° DP as a secondary procedure during gastric or colonic resection;
- ° Colonic resection required for cancer extension (gastric resection allowed);
- ° Additional hepatic resection;
- ° Participation to another study with interference with study outcome;
- ° ASA 4 / WHO 3;
- ° Arterial resection other than splenic artery.

### Indications for DP

Numerous indications for elective DP exist, and the most common indications are as follows: ductal adenocarcinoma, mucinous cystic neoplasm, intraductal papillary mucinous neoplasm, neuroendocrine tumor, solid-pseudopapillary neoplasms, chronic pancreatitis. All indications are included in this trial.

### Sample size calculation

The PANDORINA trial is as a non-inferiority randomized trial, hypothesizing that the outcome (i.e., postoperative complications Clavien-Dindo score ≥ 3 and, secondarily, clinically relevant POPF, grades B and C) in patients undergoing DP without prophylactic abdominal drainage are non-inferior to those of patients with routine prophylactic abdominal drainage after surgery. The sample size is calculated according to the formula (available at https://www.sealedenvelope.com/power/binary-noninferior/):

$$n=f\left(\alpha, \beta \right)\times \left[{\pi}_{\mathrm{s}}\times \left(100-{\pi}_{\mathrm{s}}\right)+{\pi}_{\mathrm{e}}\times \left(100-{\pi}_{\mathrm{e}}\right)\right]/{\left({\pi}_{\mathrm{s}}-{\pi}_{\mathrm{e}}-d\right)}^2$$

where *π*_s_ and *π*_e_ are the true percent “success” in the “drain” and “no drain” groups respectively.

$$F\left(\alpha, \beta \right)={\left[{\varPhi}^{-1}\left(\alpha \right)+{\varPhi}^{-1}\left(\beta \right)\right]}^2$$

And *φ*^−1^ is the cumulative distribution function of a standardized normal deviate.

Patients recruited in the PANDORINA trial will be randomized within the referred Institute with a 1:1 allocation ratio. The primary endpoint of the present trial is the rate of Clavien-Dindo ≥3 major complications, following the assumption: 2.5% one-sided significance level (*α*), 80% power (1−*β*), and a non-inferiority level of 8%. The expected success percentage in the intervention arm (no prophylactic abdominal drainage) of 77% and a success percentage in the control group of 70% (prophylactic abdominal drainage, based on the multicenter LEOPARD trial with a majority of minimally invasive procedures) [21]. The needed number of patients was calculated with a sealed envelope: a total of 272 patients are required. Including a 3% of possible drop-out after randomization, the total required sample size is 282 patients (141 patients per arm).

The most relevant secondary endpoint is the rate of clinically relevant POPF (grades B and C). The sample size was calculated with the following assumption: 2.5% one-sided significance level (*α*), 80% power (1−*β*), and a non-inferiority level of 8%. The expected success percentage in the intervention arm (no prophylactic abdominal drainage) is 81%, and a success percentage in the control group (prophylactic abdominal drainage) is 75%. This difference of 6% less grade B/C POPF without abdominal drainage is based on the Van Buren trial, and the baseline risk of 25% grade B/C POPF is based on the recently published distal fistula risk score (D-FRS), a combined Dutch/Verona multicenter study [22]. The needed number of patients was calculated with sealed envelope: a total of 274 patients. Including a 3% of possible drop-out after randomization, the total required sample size is 282 patients (141 patients per arm).

Based on the second (slightly lager) sample size calculation, we will include 282 patients so that conclusions can be drawn for both endpoints.

### Stratification

The patients included in the present trial will be stratified in preoperative estimated high and low risk for grade B/C POPF. High-risk patients is defined as a pancreatic duct diameter > 3 mm and/or pancreatic height (at the neck) > 19 mm based on the D-FRS [22]. Other patients are considered as low risk. Patients will also be stratified based on annual hospital volume of DP. Stratification will be as follows: high volume > 40 DPs annual and low volume will be ≤ 39 DPs annual.

### Quality

Participating centers should perform at least 10 DPs per year for any diagnosis. Surgeons should have performed > 50 pancreatic resections (any type, any diagnosis) in the past 5 years and > 20 DPs for any diagnosis ever. All sixteen centers participating in the Dutch Pancreatic Cancer Group are invited.

## Treatment of subjects

### Investigation treatment

#### Work-up

Preoperative work-up is according to routine practice.

#### Surgical technique

Agreement was reached regarding the surgical technique standards that should be followed during both open and minimally invasive DP. The following highlights are allowed as long as the same technique is applied both in the intervention and control group:

- Transection of the pancreas will be performed with a stapler* using the commonly used gradual stepwise compression technique (i.e., 4–5 min to close the stapler to prevent (micro-)rupture of the pancreas), as described by Asbun et al. [17], in both open and minimally invasive surgery. In all patients, graded compression technique is used as described by Asbun et al. with the same type of stapler. HJ Asbun was a senior advisor in the design of this current study.
- In case of a soft pancreas with transection at the pancreatic neck, a white (vascular) or blue stapler filling is used; in case of a fibrotic thick pancreas, a green stapler filling is used.
- Use of co-interventions for pancreatic stump closure is not advised. If they are used, such as human fibrinogen/thrombin sealant, fibrin-like glues, bio-absorbable reinforcement, autologous patches, and suture closure, they should be used routinely in all patients undergoing DP (i.e., in both arms of the trial) in that particular center;
- Preoperative endoscopic injection of botulinum toxin into the Sphincter of Oddi to prevent postoperative pancreatic fistula after DP is not advised. If used, this is not an exclusion criteria as long as it is used routinely in all patients undergoing DP (i.e., in both arms of the trial) in that particular center [23, 24].
  - ◦ *During the trial, one type of stapler is used (Ethicon®) to reduce heterogeneity.

#### Somatostatin analogs

Use of somatostatin analogs (e.g., Pasireotide®) is not advised. If used, this is not an exclusion criteria as long as it is used routinely in all patients undergoing DP (i.e., in both arms of the trial) in that particular center [25].

#### Prophylactic abdominal drainage

In the control arm, the abdominal drain will be placed intraoperatively when randomized into the drain group. In case of splenectomy, the drain is placed such that it includes the former splenic fossa with drain openings in this area and extending its tip until next to the pancreatic transection margin while avoiding direct contact with the splenic artery/vein stumps. The placed intra-abdominal drains are passive drains and will evacuate fluid by passive gravity, without active suction.

In the drain-group amylase levels are determined on day 1, 3, and 5 postoperatively (day 0 = day of DP). The drain can be removed on day 3 unless the drain levels exceed three times the upper limit of the institutions range of serum amylase (i.e., 2016 updated ISGPS definition of pancreatic fistula) or when the amount exceeds 200ml in 24h the fluid aspect is suspicious. This stands also for those patients in the no-drain group who, despite randomization, receive a percutaneous catheter drainage for any intraoperative reasons of concern.

For other details of the surgical procedure, surgeons can use a surgical technique according to his/her own preference as long as they use the same approach in all patients. All procedure details should be recorded within the case report form.

### General treatment regimen

Postoperative care is similar in both arms and based on enhanced recovery principles, which include early mobilization and expanding oral intake as desired by the patient. In the following sub-sections, we will clarify the recommendations for perioperative care. In case of radiological percutaneous drainage, amylase and culture must be determined in the drain fluid.

### Use of co-intervention

There are no specific co-interventions.

## Methods

### Definitions

All definitions are displayed in the Additional file 1. The left pancreas is defined as the pancreatic portion (body and tail) located on the left side of the porto-mesenteric vein. Complications are classified according to the Clavien-Dindo score ≥ 3 [26]. Grade B/C POPF [19], delayed gastric emptying (DGE) [27], and postpancreatectomy hemorrhage (PPH) [18] are classified using the ISGPS definitions. Surgical site infection (SSI) is classified according to the Center for Disease Control and Prevention definition [28].

### Study parameters/endpoints

#### Main parameters/endpoint

Primary endpoint is the rate of Clavien-Dindo score ≥ 3 complications [26].

#### Secondary parameters/endpoint

Secondary outcomes are grade B/C POPF, reoperation, catheter drainage, abdominal collections, wound infection, DGE, PPH, blood transfusion, length of stay (LOS), in-hospital mortality, 90-day mortality, readmission within 90 days, and start of adjuvant chemotherapy. Outcomes will be based on the Dutch Pancreatic Cancer Audit registry.

- Intraoperative parameters:
  - ° Date of operation;
  - ° Splenectomy;
  - ° Conversion;
  - ° Reason for conversion;
  - ° Vessel resection (excluding splenic vessels); if so, type of venous resection/reconstruction;
  - ° Operative time (from first incision to closure of the abdomen), minutes;
  - ° Intraoperative blood loss, mL (suction canister and weight of gauzes);
  - ° Intraoperative blood transfusion, mL fresh frozen plasma or packed cells.
- Postoperative parameters:
  - ° POPF (grade B/C)*;
  - ° Major complications (CD ≥ 3);
  - ° DGE (grade B/C)*;
  - ° PPH (grade B/C)*;
  - ° SSI*;
  - ° Postoperative intervention (radiology, endoscopy, surgery);
  - ° Reason for postoperative intervention.
- Hospitalization parameters:
  - ° LOS, days;
  - ° Readmission;
  - ° Intensive care admission;
  - ° Reason for intensive care admission;
  - ° Duration of intensive care admission, days;
  - ° CRP on 3rd postoperative day;
  - ° Amount of days with drain in place.
- See Additional file 1 for detailed definitions of study outcomes.

#### Other study parameters

Other study parameters are baseline characteristics, including:

- Patient study identification number;
- Date of birth;
- Sex;
- Performance status (Karnofsky score)*;
- ASA physical status*;
- Body mass index (BMI), kg/m^2)^;
- Abdominal surgery in history;
- Diabetes mellitus;
- Diagnosis based on preoperative imaging.

*See Additional file 1 for detailed definitions of study outcomes.

### Randomization and treatment allocation

Eligible patients for the study will be identified at the outpatient clinic and in this stage informed consent will be obtained; the randomization will take place directly after start of the operation, when the decision is made to proceed with the resection. The surgeon performing the operation will call the study coordinator to perform the randomization in Castor EDC. Patients will be analyzed according to the allocated treatment, as per intention-to-treat principles. If during the operation is chosen not to act conform the randomization, specification of this choice is required. A per-protocol analysis will also be performed for the primary endpoint.

A data safety monitoring committee will assess safety endpoints per 50 randomized patients. All patients will be randomized centrally using an online computer-controlled permuted-block randomization module in a 1:1 ratio. The block sizes itself will be subject to random variation with block sizes varying from 4 to 8 patients. The entire randomization will be concealed to all involved investigators except the trial coordinators. Patients will be coded by a numeric randomization code and the principal investigator will be the only one with access to it and investigators and patients are not blinded. Patients will be stratified according to high or low risk and minimally invasive and open procedure. The source data will be stored digitally and will be kept by the project leader for 15 years after the inclusion of the last patient.

### Study procedures

#### Patient screening procedures

Screening of patients consists of standard procedures, including high-quality computed tomography (CT) of the pancreas. Screening procedures will be according to the local treating team’s preference.

#### Data collection

Baseline characteristics will be recorded before randomization. The required clinical data, i.e., primary and secondary outcomes, will be collected after randomization, i.e., from hospitalization up to 6 months postoperatively using standardized case report forms, and in Dutch centers according to the mandatory Dutch Pancreatic Cancer Audit which is coded. All complications will be scored using the Clavien-Dindo score of surgical complications (see Additional file 1).

#### Registration of patients not included in the PANDORINA trial

All patients undergoing elective DP but not included in the trial will be logged using a screening log. These patients will be registered anonymously using a standardized prospectively collected database. Due to this, it will be possible to assess the presence of patient selection by indication.

### Withdrawal of individual subjects

Subjects can withdraw from the study at any time for any reason if they wish to do so without any consequences. The investigator can decide to withdraw a subject from the study for urgent medical reasons.

### Replacement of individual subjects after withdrawal

Patients withdrawn after surgery will not be replaced. If a patient does not receive a resection due to intraoperative metastasis or other reasons for not performing a DP, he will not be randomized since the randomization process takes place only after the intention to resect. Patients withdrawn because of treatment-related reasons will not be replaced.

### Follow-up of subjects withdrawn from treatment

All subjects will be analyzed according to intention-to-treat principles and follow-up will be 6 months after DP.

## Safety reporting

### Safety reporting in the Netherlands

Safety reporting may be varying between participating centers or different countries. Sections below describe the details of safety reporting for Dutch medical centers.

#### Section 10 WMO event

In accordance to section 10, subsection 1, of the WMO, the investigator will inform the subjects and the reviewing accredited MEC if anything occurs, on the basis of which it appears that the disadvantages of participation may be significantly greater than was foreseen in the research proposal. The study will be suspended pending further review by the accredited Medical Ethics Review Committee, except insofar as suspension would jeopardize the subjects’ health. The investigator will take care that all subjects are kept informed.

#### AEs and SAEs

##### Adverse events (AEs)

Adverse events are defined as any undesirable experience occurring to a subject during the study, whether or not considered related to the placement of a drain after DP or not. All adverse events reported spontaneously by the subject or observed by the investigator or his staff will be recorded.

##### Serious adverse events (SAEs)

A serious adverse event is any untoward medical occurrence or effect that at any dose:

- Results in death;
- Is life threatening (at the time of the event);
- Requires hospitalization or prolongation of existing inpatients’ hospitalization;
- Results in persistent or significant disability or incapacity;
- Any other important medical event that may not result in death, be life threatening, or require hospitalization, may be considered a serious adverse experience when, based upon appropriate medical judgment, the event may jeopardize the subject or may require an intervention to prevent one of the outcomes listed above.

Predefined list of SAEs that will be reported to the study coordinator and CCMO:

- SAE resulting in mortality (for any reason);
- SAE necessitating surgical reintervention;
- SAE resulting in ICU admission.

The sponsor will report these SAEs through the preferred web portal to the accredited MEC that approved the protocol, within 15 days after the sponsor has first knowledge of the serious adverse events. SAEs that result in death or are life threatening will be reported expeditely. The expedited reporting will occur not later than 7 days after the responsible investigator has first knowledge of the adverse event. This is for a preliminary report with another 8 days for completion of the report. All SAEs will be reported until end of study. All participating physicians involved in the PANDORINA trial have to brief the study coordinator in case of mortality or any unexpected event that leads to a prolongation of the hospitalization or to readmission. Primary and secondary outcomes do not have to be reported immediately to the study coordinator. Mortality has to be reported by the attending physician to the study coordinator within 48 h after the occurrence.

The following SAEs are recorded in an overview list that will be submitted once a year to the MEC of the Amsterdam UMC, Amsterdam, the Netherlands. If mandatory, these SAEs are also reported to MECs of participating centers. These SAEs include, among others:

- Readmission;
- POPF grades B and C;
- PPH grades B and C;
- DGE grades B and C;
- Percutaneous catheter drainage.

#### Follow-up of adverse event

Follow-up for AEs will be at least up to 30 days after treatment. All AEs will be followed until they have abated, or until a stable situation has been reached. Depending on the event, follow-up may require additional tests or medical procedures as indicated, and/or referral to the general physician or a medical specialist. Common (serious) adverse events after pancreatic surgery, such as POPF, DGH and PPH will not be reported, as these complications occur in a large subset (>15–25%) of patients. Other adverse events will be reported until end of study, as defined in the protocol.

#### Data safety monitoring board

An independent data safety monitoring board (DSMB) will be appointed to evaluate the study safety parameters (all patients). After every 50 included patients, when 1 month of follow-up of these patients has passed, the DSMB will have a meeting and assess the safety parameters. This meeting may be a telephone or video conference. This DSMB will exist of one independent statisticians and two independent surgeons. One surgeon will be appointed as the DSMB chairman and a second member will act as secretary. The minutes of this meeting will be sent to the sponsor of the study by the study coordinator. The DSMB will not be blinded and fully informed on all SAEs. The DSMB can request a full report of specific study outcomes whenever required. The study coordinator and principal investigator will only be present during the start of the DSMB meeting to provide the data and provide background information. The result of the DSMB meeting will be sent to the trial steering committee, the sponsor of the study, and the Research Ethics Committee. Should the sponsor decide not to fully implement the advice of the DSMB, the authors will have to motivate their discussion for the Research Ethics Committee.

#### Study monitoring

Only in centers requiring such monitoring, a clinical research associate will monitor the study. All monitoring visitations will be scheduled at mutually agreeable times, periodically during the study at a frequency deemed appropriate. These visits will be conducted to evaluate the progress of the study, to ensure that the rights and well-being of the subjects are protected, to check that the reported clinical study data are accurate, complete, and verifiable from source documents, and if the conduct of the study is in compliance with the approved protocol and amendments, good clinical practice, and applicable national regulatory requirements. A monitoring visitation will include a review of the essential clinical study documents (regulatory documents, case report forms, source documents, subject informed consent forms, etc.) as well as discussion on the conduct of the study with the investigators. The investigators should be available during these visitations to facilitate the review of the clinical study records and to discuss, resolve, and document any discrepancies found during the visitation. Auditing will be performed at 50% of the inclusions and at the end of the study. The Trial Steering Group will review data at 50% of the inclusions.

## Statistical analysis

Primary and secondary endpoints will be cross checked with data from primary sources and a blinded adjudication committee will check them against the definitions, which are established before the start of this study. Frequencies will be presented for dichotomous data. The primary endpoint Clavien-Dindo score ≥ 3 complications will be tested for non-inferiority using the chi-square test. The distribution of variables will be determined using several plots (boxplot, Q-Q plot, and histogram) and the Kolmogorov-Smirnov, Shapiro-Wilk, and Levene’s tests. For comparison of normally distributed continuous variables, the independent samples *t*-test will be used and values will be expressed as means (standard deviations). Continuous non-normally distributed variables will be compared using the Mann-Whitney *U* test and values will be expressed as medians (interquartile ranges). Categorical variables will be compared by chi-square or Fisher’s exact test as appropriate, and values will be expressed as proportions. A two-tailed *p*-value <0.05 will be considered statistically significant. Where possible, risk ratios with 95% confidence intervals will be reported. For the primary study outcome, a two-sided 95% confidence interval will be reported. Time to event endpoints, such as survival, will be calculated using Kaplan-Meier estimations. A Cox regression analysis will be performed to investigate predictors of postoperative survival. All parameters with a *p*-value <0.1 in a univariable analysis are included in the multivariable Cox regression analysis. Additionally, multivariable analyses are performed to determine predictors for primary and secondary study outcomes, for example R0 resection, the occurrence of postoperative pancreatic fistula. Intraoperative details and primary endpoint of this study are expected to be complete. All data entries should be sent to the project leader immediately after the final examination. In case of missing data, explanation should be given to the project leader. For subjects who are lost to follow-up, a sensitivity analysis will be performed to determine best-case/worst-case scenarios. A detailed statistical analysis plan will be drafted prior to database lock. Despite all prior preventive measures taken, a complex multinational trial may still evoke unforeseen situations after database lock that threaten data integrity and can only be resolved by unlocking the database prior to the final analysis. For purpose of transparency and reproducibility, the statistical analysis plan will therefore also describe the procedure to be followed when such situations arise.

## Ethical considerations

### Regulation statement

The PANDORINA trial will be conducted according to the principles of the Declaration of Helsinki (64th version, October 2013) and in accordance with the local laws and regulations, such as in the Netherlands the Medical Research Involving Human Subjects Act. The local principal investigator is responsible for making sure that local laws and regulations are followed.

### Recruitment and consent

All subjects will be recruited at the outpatient clinic by one of the principal investigators. The principal investigator may be replaced by an assigned substitute, who is fully informed and aware of the study requirements and procedures, e.g., the study coordinator, local treating physician, or a study nurse. The approached patient will be given at least 24 hours’ time to consider informed consent and to make a decision. If indicated, surgery will be performed within 4 weeks after determination of the diagnosis, so there is sufficient time for explanation of the disease severity. Furthermore, due to this the patient will have sufficient time to explain her/his prospects and to consider the (dis)advantages of participating in the study. Participant retention and complete follow-up was promoted by periodic meetings and site visits with outcome data and corresponding time windows.

### Objections by minors or incapacitated subjects

Minors and incapacitated patients will not be included in this study.

### Benefits and risks assessment group

Prophylactic abdominal drainage can be useful for early detection of pancreatic juice in case of a POPF but drains might themselves also contribute to these complications. Patients will not undergo additional investigations and interventions due to participation in the PANDORINA trial and therefore risks to subjects involved in this trial are similar to every other patient undergoing DP in routine clinical practice, especially since the Van Buren multicenter RCT already demonstrated the safety of omitting prophylactic abdominal drainage. Potential benefits for subjects in the investigational treatment arm could be fewer major complications, less abdominal pain, and less discomfort in case of no prophylactic drainage.

## Public disclosure and publication policy

No arrangements have been made concerning public disclosure and publication of the research data and outcomes. The trial was registered within Netherlands Trial Register (https://www.trialregister.nl/trial/9116) with trial number NL9116 on 11-12-2020. The results of this trial will be submitted to a high-impact peer-reviewed medical journal regardless of the study outcome. Authorship will be based on the most recent international ICMJE guidelines. Next to these ICMJE guidelines, a minimum of 5 randomized patients is required for 1 co-authorship per participating center, a minimum of 20 randomized patients for 2 co-authorships and a minimum 40 randomized patients for 3 authorships. Per site, it is internally determined which local investigator will be author as long as this person fulfils the international ICMJE guidelines for authorship.

The study PhD coordinators will be the first authors. There is one trial coordinator responsible for the coordination of the trial for all Dutch centers (EAVB) and one trial coordinator for all Italian centers (AB). This includes recruitment, randomization, and follow-up of the study subjects as well as communication with the study sites. The penultimate and last authorships are for the three principal investigators (CB, CvE, MGB). All other authors will be listed in alphabetical order. Clinicians who are involved in this study and do not fulfil the previously mentioned authorship criteria will be listed as “collaborator” in the final manuscript and the medical journal will be asked to present the names of these collaborators in PubMed. For purposes protocol amendment, abstract presentation, and publication, any secondary publication will be delegated to the appropriate principal authors.

## Discussion

PANDORINA is the first binational, multicenter, randomized controlled non-inferiority trial with the primary objective to evaluate the hypothesis that omitting prophylactic abdominal drainage after DP does not worsen the risk of postoperative severe complications [18, 19]. The focus of this study is to assess if operative placed drains lead to less complicated POPF which need a reintervention or reoperation. However, POPF B/C without intervention cannot be measured in the no drain arm of our study since this is because of prolonged operative drainage. Therefore, the primary endpoint is chosen to be severe morbidity, while this is caused by a POPF grade B/C, on which the clinical focus will lie. Most of the published studies on drain placement after pancreatectomy focus on both pancreatoduodenectomy and DP, but these two entities present are associated with different complications and therefore deserve separate evaluation [29, 30]. The PANDORINA trial is innovative since it takes the preoperative risk on POPF into account based on the D-FRS and it warrants homogenous stump closing by using the same graded compression technique and same stapling devive [22, 24].

Several studies provide better insight into the value of abdominal drainage after PD. In a cohort of 69 patients after DP (39 with and 30 without a drain), Paulus et al. [31] did not observe a decrease in the incidence of intra-abdominal abscess, POPF [32], or pseudocyst formation in case of intra-abdominal drainage. In a randomized trial by Conlon et al [1] in 179 patients undergoing pancreatic resection (among them 40 distal pancreatectomies), no benefit was seen for prophylactic abdominal drainage*.* However, the statistical analysis was conducted without distinction between pancreatoduodenectomy and DP. Behrman et al. [6] used propensity scored matching to compare 706 patients undergoing DP with or without prophylactic abdominal drainage. They observed a higher incidence of POPF in those patients who received drains; however, their analysis included the clinically non-relevant POPF Grade A [33].

A randomized trial by Bassi et al. reported the outcome of early vs late removal of the intra-abdominal drain after pancreatectomy in 114 patients of whom 39 underwent DP [33]. They found an increase incidence of POPF, intra-abdominal infected collections, pulmonary complications, length of stay (LOS), and readmission in the group of late drain removal. They concluded that late drain removal is a risk factor for POPF. This finding was confirmed in a recent study by Seykora et al. in which in 5581 DPs (POPF grade B/C rate 17%) early drain removal (in 716 patients) was associated with improved outcomes [34].

Recently, Van Buren et al. reported the first multicenter randomized controlled trial in 344 patients undergoing DP with (*n*=174) and without (*n*=170) prophylactic intra-abdominal drainage [2]. Their hypothesis was that DP without routine intra-abdominal drainage does not affect the frequency of grade 2 or higher-grade complication (according to the Common Terminology Criteria for Adverse Event [23]). The rate of grade 2 complications was comparable between the groups (44% vs. 42%, *p*=0.80). The rate of grade B/C POPF did not differ and was 6% lower in the group without abdominal drainage (18% vs 12%, *p*=0.11) either. This led to the conclusion that clinical outcomes after DP with or without drain are comparable. The Van Buren trial does not describe a standardized technique of pancreatic stump closure and does not stratify patients to their risk of POPF after DP.

Asbun et al. described the now commonly used graded compression technique for DP [17, 24]. By compressing the pancreatic tissue gradually based on the experienced resistance, the pancreatic parenchyma is compressed rather than crushed before transecting, which results in low rates of POPF [17]. A reduction in POPF using this technique was confirmed in two other studies, one study showed that out of 42 patients undergoing DP, 17 were treated with the graded compression technique and did not develop POPF (0%) compared to 28% of the patients in the comparison group [35, 36]. The intra-abdominal drainage period and the median LOS were significantly shorter in the graded compression group compared to the no-graded compression group.

In order to assess the rationale of prophylactic abdominal drainage after DP, we designed a binational multicenter randomized control trial (RCT). All patients undergoing DP with open and minimally invasive techniques (laparoscopic and robotic), with and without preservation of the spleen, are eligible for the study.

## Conclusion

PANDORINA is a binational multicenter, randomized controlled non-inferiority trial with the primary objective to evaluate that omitting prophylactic abdominal drainage after DP does not worsen the risk of postoperative Clavien-Dindo score ≥ 3 complications [18, 19]. Additional to the current literature, the PANDORINA trial makes prespecified fistula risk groups and guarantees homogenous stump closure.

### Trial status

Confirmation of funding of the trial by Ethicon UK (Johnson & Johnson Medical Limited, Edinburgh, UK) was received on September 25, 2017. Ethical approval in the Amsterdam UMC was received on September 25, 2020. The PANDORINA trial was registered in the Netherlands Trial Register on December 11, 2020 (NL9116). The first patient was randomized on November 02, 2020. At the time of submitting this protocol for publication (May 4, 2022), all centers were actively recruiting patients for the trial and 152 out of 282 (54%) have been randomized, which means that inclusion is on schedule.

## Supplementary Information

**Additional file 1.**

## Data Availability

Data will be available to the study coordinators. All publications and presentation using this data will be approved by all authors.
