# Correction: Prophylactic abdominal drainage or no drainage after distal pancreatectomy (PANDORINA): a study protocol of a binational multicenter randomized controlled trial

**DOI:** 10.1186/s13063-022-06957-8

**Published:** 2023-02-20

**Authors:** F. L. Vissers, A. Balduzzi, E. A. van Bodegraven, J. van Hilst, S. Festen, M. Abu Hilal, H. J. Asbun, J. S. D. Mieog, B. Groot Koerkamp, O. R. Busch, F. Daams, M. Luyer, M. De Pastena, G. Malleo, G. Marchegiani, J. Klaase, I. Q. Molenaar, R. Salvia, H. C. van Santvoort, M. Stommel, D. Lips, M. Coolsen, C. Bassi, C. van Eijck, M. G. Besselink, for the Dutch Pancreatic Cancer Group

**Affiliations:** 1 Department of Surgery, Amsterdam UMC, location University of Amsterdam, Amsterdam, the Netherlands; 2 Cancer Center Amsterdam, Amsterdam, the Netherlands; 3 Department of Surgery, Pancreas Institute, Verona University Hospital, Verona, Italy; 4 Department of Surgery, OLVG, Amsterdam, the Netherlands; 5 Department of Surgery, University Hospital Southampton NHS Foundation Trust, Southampton, UK; 6 Department of Surgery, Poliambulanza Hospital Brescia, Brescia, Italy; 7 Division of Hepatobiliary and Pancreas Surgery, Miami Cancer Institute, Miami, USA; 8 Department of Surgery, LUMC, Leiden, the Netherlands; 9 Department of Surgery, Erasmus MC, Rotterdam, the Netherlands; 10 Department of Surgery, Catharina Hospital, Eindhoven, the Netherlands; 11 Department of Surgery, University Medical Center Groningen, Groningen, the Netherlands; 12 Department of Surgery, Regional Academic Cancer Center Utrecht, University Medical Center Utrecht, Utrecht, the Netherlands; 13 Department of Surgery, Regional Academic Cancer Center Utrecht, St Antonius Hospital Nieuwegein, Utrecht, the Netherlands; 14 Department of Surgery, Radboud UMC, Nijmegen, the Netherlands; 15 Department of Surgery, Medisch Spectrum Twente, Enschede, the Netherlands; 16 Department of Surgery, Maastricht Universitair Medisch Centrum, Maastricht, the Netherlands

**Correction: Trials 23, 809 (2022)**

**https://doi.org/10.1186/s13063-022-06736-5**

Error 1 (Title) [1]

The sentence currently reads:

“Prophylactic abdominal drainage or no drainage after distal pancreatectomy (PANDORINA): a binational multicenter randomized controlled trial”

The sentence needs to read:

“Prophylactic abdominal drainage or no drainage after distal pancreatectomy (PANDORINA): a study protocol of a binational multicenter randomized controlled trial”

Error 2 (Abstract)

The sentence currently reads:

“Routine drainage after DP could potentially be omitted and this could even be beneficial because of the hypothetical prevention of drain-induced infections (Fisher, Surgery 52:205-22, 2018).”

The sentence needs to read:

“Routine drainage after DP could potentially be omitted and this could even be beneficial because of the hypothetical prevention of drain-induced infections (Fisher, 2018).”
