# Supplementary figures and images for: Prophylactic abdominal drainage or no drainage after distal pancreatectomy (PANDORINA): a binational multicenter randomized controlled trial
Source: Trials. 2022 Sep 24;23:809. doi: 10.1186/s13063-022-06736-5 (PMC9509576; doi:10.1186/s13063-022-06736-5)

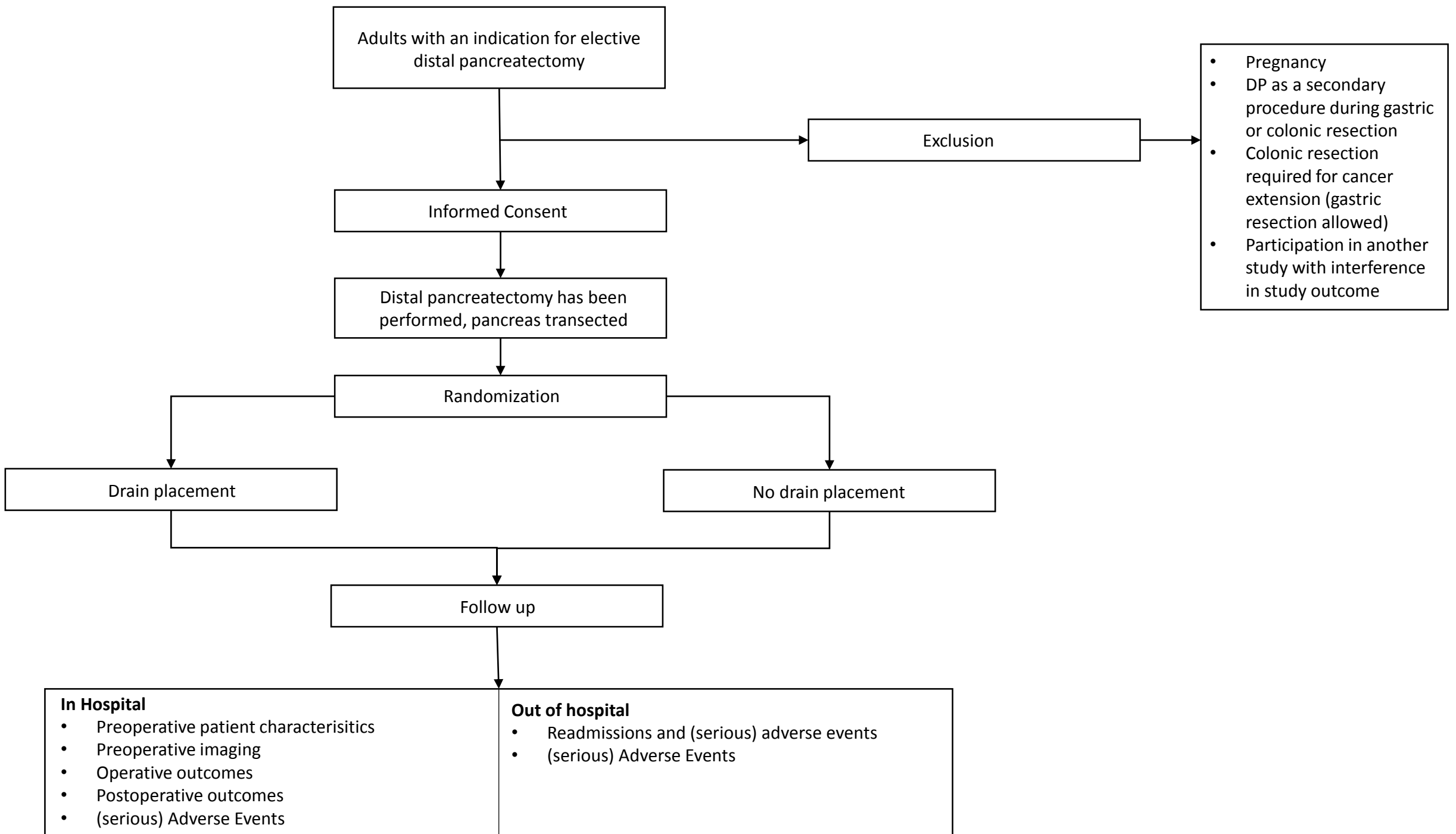

Supplement: Supplementary file 1 — Additional file 1. [file 13063_2022_6736_MOESM1_ESM.pdf]
